# Distal outcomes in mixture modeling: A guide for pairwise comparisons, multiplicity control, and effect size reporting

**DOI:** 10.3758/s13428-026-03144-4

**Published:** 2026-08-14

**Authors:** Delwin B. Carter

**Affiliations:** Gevirtz Graduate School of Education, University of California, Santa Barbara, Santa Barbara, CA 93106 USA

**Keywords:** Mixture modeling, Distal outcomes, Latent class analysis, Auxiliary variables, Multiple comparisons, Effect sizes

## Abstract

Despite the widespread use of mixture models in psychology, education, and the behavioral sciences, there is little consolidated guidance on how distal outcome differences should be tested, corrected for multiplicity, and reported once a latent class or profile solution has been selected. In practice, applied researchers often report omnibus tests without follow-up comparisons, conduct uncorrected pairwise tests, or omit effect sizes and confidence intervals altogether, limiting the interpretability and reproducibility of findin**gs.** Consistent with this concern, a targeted reporting-practice audit of recent applied person-centered studies showed that multiplicity adjustment, global distal outcome effect sizes, pairwise effect sizes, and confidence intervals for pairwise effects were rarely reported. The present paper synthesizes recommendations from the general statistical literature and adapts them to the context of mixture modeling, focusing primarily on continuous distal outcomes and comparisons of class-specific means. We propose a principled framework for (a) defining appropriate families of pairwise comparisons for distal outcomes, (b) selecting and implementing multiplicity corrections with Benjamini–Hochberg recommended as the default procedure for applied distal outcome comparisons, and (c) computing and reporting global and pairwise effect sizes and confidence intervals using quantities readily available from standard mixture modeling software (e.g., Mplus). Through worked examples and a software-agnostic Quarto/R supplement, we demonstrate how these practices can be implemented transparently and consistently across common auxiliary-variable approaches, including maximum likelihood (ML) three-step and Bolck–Croon–Hagenaars (BCH) methods.

**Supplementary Information:**

The online version contains supplementary material available at https://doi.org/10.3758/s13428-026-03144-4.

Mixture modeling is widely used across the behavioral sciences to examine unobserved population heterogeneity by identifying latent classes or profiles based on patterns of responses to observed indicators (McLachlan & Peel, 2000). Unlike continuous latent variable models, mixture models posit a categorical latent variable that partitions individuals into distinct subgroups (Nylund-Gibson & Choi, 2018). After selecting a measurement model, researchers commonly extend these models to address substantive research questions by relating class membership to external variables, including covariates and distal outcomes. In applied research, differences on distal outcomes are often central to substantive interpretation and are frequently used to justify theoretical conclusions or policy-relevant decisions.

A primary goal of applied mixture modeling research is to evaluate whether latent classes or profiles differ meaningfully on external outcome variables, commonly referred to as distal outcomes (Nylund-Gibson et al., 2019). Such comparisons are routinely used to characterize the substantive meaning of identified subgroups and to motivate theoretical or policy-relevant conclusions. For example, prior studies have examined class differences across multiple psychological, academic, and health-related outcomes, often involving several latent classes and numerous pairwise comparisons among class-specific means (e.g., Boutin-Martinez et al., 2019; Felix et al., 2019; Kam et al., 2021; Moore et al., 2024). In these settings, distal outcome analyses frequently involve dozens of statistical tests conducted within a single study, amplifying the risk of inflated Type I error and complicating interpretation when results are reported without explicit correction, effect size estimates, or confidence intervals.

Accordingly, the present paper focuses primarily on continuous distal outcomes and comparisons of class-specific means. Although some general principles may extend to categorical outcomes, those outcomes require additional inferential and effect-size considerations beyond the scope of the present workflow.

## Auxiliary variable approaches in mixture models

Distal outcome differences can be examined following class or profile enumeration using a variety of auxiliary-variable approaches, regardless of the specific mixture modeling framework employed (e.g., latent class analysis [LCA], latent profile analysis [LPA], growth mixture modeling [GMM]; Muthén & Asparouhov, 2014). Contemporary recommendations emphasize stepwise estimation procedures, in which the measurement model is first identified and subsequently held fixed while relationships with external variables are evaluated (Vermunt, 2010; Bakk et al., 2013; Nylund-Gibson et al., 2019). These approaches are preferred because jointly estimating measurement and structural components can alter class formation and compromise interpretability. Among the available methods, the maximum likelihood (ML) three-step and Bolck–Croon–Hagenaars (BCH) approaches are now widely used in applied research and provide standard outputs for testing distal outcome differences. Importantly, regardless of the auxiliary-variable method employed, researchers face the same inferential challenges when conducting and reporting multiple distal outcome comparisons—challenges that are the focus of the present paper.

### ML 3-step

The ML three-step approach (Vermunt, 2010) is a widely used method for relating latent class or profile membership to external variables while minimizing distortion of the measurement model. In practice, both automated and manual implementations are available, with the latter allowing for the inclusion of covariates and distal outcomes within a stepwise framework. Across implementations, distal outcome analyses under the ML three-step approach yield omnibus Wald tests and class-specific mean estimates that are commonly followed by pairwise comparisons among classes. As a result, studies employing the ML three-step method frequently involve multiple hypothesis tests that require careful inferential control.

### Bolck, Croon, & Hagenaars (BCH) method

The BCH approach (Bolck et al., 2004; Bakk et al., 2013; Asparouhov & Muthén, 2020) provides an alternative auxiliary-variable framework for evaluating distal outcome differences following mixture model estimation. Similar to the ML three-step approach, both automated and manual implementations of BCH are available and are commonly used in applied research. Regardless of implementation, BCH-based distal outcome analyses yield class-specific mean estimates and omnibus Wald tests that are often followed by multiple pairwise comparisons. Consequently, studies using the BCH method are subject to the same challenges related to multiplicity, effect size estimation, and transparent reporting that arise under other auxiliary-variable approaches.

Although the inferential issues addressed in this paper overlap with broader concerns in post hoc testing and multiple comparisons, distal outcome analyses in mixture modeling present a distinct reporting context. Unlike observed-group comparisons, latent classes and profiles are model-estimated subgroups, and distal outcome means are typically obtained through auxiliary-variable procedures designed to preserve the measurement model. As a result, applied researchers must translate mixture model output, often including class-specific estimates, Wald tests, pairwise comparison results, and classification-adjusted quantities, into a coherent reporting framework. This translation is not automatic. Researchers must decide how to define the family of comparisons, whether and how to adjust pairwise tests for multiplicity, how to quantify the global magnitude of distal outcome separation, and how to report the magnitude and precision of specific class-to-class differences.

When distal outcomes are included in mixture models, differences in class-specific outcome means should first be evaluated using an omnibus test of equality prior to conducting pairwise comparisons. In practice, this is typically implemented as a Wald test assessing whether all class-specific distal outcome means are equal (Asparouhov, 2007). Although mixture modeling software such as Mplus (Muthén & Muthén, 1998–2017) provides the necessary test statistics, omnibus tests are not always automatically requested or clearly labeled in output files, and applied researchers may inadvertently proceed directly to pairwise comparisons without first establishing evidence of an overall class effect. Explicitly requesting, reporting, and interpreting an omnibus test is therefore a critical first step in distal outcome inference, as it defines the family of subsequent pairwise comparisons and provides the appropriate context for multiplicity-adjusted follow-up testing.

To clarify the recommended sequence of analytic and reporting decisions for distal outcome analyses in mixture models, Table 1 summarizes a step-by-step workflow that integrates omnibus testing, multiplicity control, effect size estimation, and confidence interval reporting. This workflow is method-agnostic with respect to the specific mixture modeling framework (e.g., ML three-step or BCH) and is intended to support transparent, reproducible, and interpretable reporting of distal outcome results by making the ordering of analytic decisions explicit.

**Table 1:** Recommended workflow for distal outcome analyses in mixture models

Step	Analytic stage	Purpose	Typical output
1	Estimate measurement model	Identify latent class or profile solution independent of distal outcomes	Class solution, class proportions
2	Include distal outcome using a stepwise approach (e.g., ML 3-step or BCH)	Preserve the measurement model while estimating distal outcome parameters	Class-specific distal means
3	Conduct omnibus test of distal outcome differences (e.g., Wald test)	Assess whether any class differences exist prior to follow-up testing	Overall test statistic and *p*-value
4	Conduct pairwise comparisons across classes	Identify specific class-to-class differences	Pairwise mean differences
5	Apply multiplicity correction to pairwise tests	Control error rates across the family of comparisons	Adjusted significance decisions
6	Compute global effect size (e.g., LTB-ω)	Quantify overall association between class membership and distal outcome	Standardized global effect size
7	Compute pairwise effect sizes (e.g., Cohen’s *d*)	Quantify magnitude of specific class differences	Standardized mean differences
8	Report confidence intervals for estimates	Convey precision and uncertainty of effects	Confidence intervals for means and effect sizes

## Implementation of the reporting workflow

To support implementation of the workflow summarized in Table 1, Supplement D provides a reproducible, software-agnostic Quarto/R workflow for post-estimation distal outcome reporting. The supplement is designed for researchers who have already estimated a mixture model and obtained distal outcome results from their preferred software. Rather than requiring a specific estimation package, the workflow begins from quantities commonly available in mixture model output, including class-specific means, variances or standard deviations, class proportions or sample sizes, omnibus test information, and pairwise comparison results. It then demonstrates how to compute the reporting quantities emphasized in this tutorial, including LTB-ω, pairwise standardized effects, confidence intervals, and multiplicity-adjusted pairwise inference.

## Controlling multiplicity in distal outcome comparisons

Concerns regarding the reliability and interpretability of published findings have prompted increased attention to statistical reporting practices across the behavioral sciences (Open Science Collaboration, 2015; Wasserstein & Lazar, 2016). In response, methodological guidance has emphasized the limitations of sole reliance on null-hypothesis significance testing and the importance of supplementing *p*-values with multiplicity control, effect size estimates, and confidence intervals (Appelbaum et al., 2018; Benjamini & Hochberg, 1995; Holm, 1979; Lee, 2016). These recommendations are particularly relevant in analytic settings that involve large numbers of related hypothesis tests, where uncorrected inference can lead to inflated Type I error and over-interpretation. Despite this broader consensus, such practices have not been consistently adopted in applied mixture modeling research, especially in the context of distal outcome comparisons involving multiple latent classes.

## Targeted reporting-practice audit

To contextualize the practical need for the reporting workflow proposed in this tutorial, we conducted a targeted reporting-practice audit of recent applied person-centered studies. This audit was not designed as a meta-analysis or comprehensive systematic review; rather, it was intended to evaluate whether the specific reporting elements emphasized here were present in recent studies comparing continuous distal or external outcome means across latent groups. The final PsycINFO search returned 26 records, of which 12 met inclusion criteria after title/abstract and full-text screening. Studies were retained if they compared continuous distal or external outcome means across latent classes, profiles, trajectories, or transitions (Nylund-Gibson et al., 2023). Studies were excluded if they examined predictors of class or profile membership only, used categorical or binary distal outcomes, included outcomes as profile indicators rather than post-estimation distal outcomes, used regression models with class membership as a predictor rather than distal mean comparisons, or were methodological, simulation, tutorial, or class-enumeration-only papers.

Among the 12 retained studies, 10 reported an omnibus distal outcome test, but only two reported an adjustment for multiple pairwise comparisons. None reported LTB-ω or another global distal outcome effect size, none reported confidence intervals for pairwise effect sizes, and only one reported pairwise effect sizes. Table 2 summarizes these reporting patterns. These counts should be interpreted as a diagnostic snapshot rather than prevalence estimates for the broader literature. They nevertheless show that the reporting elements addressed in this tutorial are often absent even in recent applied studies that conduct distal outcome comparisons.

**Table 2:** Reporting elements observed in the targeted reporting-practice audit

Reporting element	Retained studies reporting element
Omnibus distal outcome test	10/12
Pairwise *p*-value adjustment	2/12
Global distal outcome effect size	0/12
Pairwise effect sizes	1/12
Confidence intervals for pairwise effect sizes	0/12

*Note.* The audit was designed as a targeted diagnostic snapshot of recent applied person-centered studies comparing continuous distal or external outcome means across latent groups. Counts should not be interpreted as prevalence estimates for the broader mixture modeling literature

These findings reinforce the need for a post-estimation reporting workflow that helps applied researchers move beyond statistical significance by reporting the magnitude, multiplicity, and precision of distal outcome differences across latent groups.

## The present paper

Regardless of the estimation approach used, mixture models typically yield latent categorical variables consisting of multiple classes or profiles, making distal outcome analysis inherently a multiple-comparisons problem. Standard practice therefore involves testing omnibus differences across classes—commonly via Wald tests—followed by pairwise comparisons of class-specific outcome means. Although similar inferential challenges have been widely discussed in the general statistical literature, including concerns about overreliance on *p*-values, inflated Type I error under multiple testing, and the omission of effect sizes and confidence intervals (Appelbaum et al., 2018; Ioannidis, 2005, 2017; Wasserstein & Lazar, 2016), corresponding guidance tailored to the post-estimation reporting demands of mixture modeling remains limited.

The present paper addresses this gap by providing a method-agnostic reporting workflow for distal outcome analyses in mixture models. The contribution is not the introduction of new omnibus or pairwise test statistics, but the organization of existing inferential tools into a practical reporting framework tailored to latent-group comparisons. Specifically, we provide guidance for (a) defining appropriate families of pairwise comparisons, (b) selecting and implementing multiplicity corrections for distal outcome tests, (c) computing and reporting a global distal outcome effect size, and (d) reporting pairwise standardized effects and confidence intervals using quantities available from standard mixture modeling output.

To lower the practical barrier to implementation, we also provide a reproducible, software-agnostic Quarto/R workflow in Supplement D. This workflow does not require researchers to estimate the mixture model in R. Instead, it begins from post-estimation quantities commonly available from mixture modeling software, including class-specific means, variances or standard deviations, class proportions or sample sizes, omnibus test information, and pairwise comparison results. The supplement then demonstrates how these quantities can be used to compute and report the core elements emphasized in this tutorial, including LTB-ω, pairwise standardized effects, confidence intervals, and multiplicity-adjusted inference. By articulating clear reporting practices and providing an executable implementation workflow, this paper aims to improve the transparency, interpretability, and reproducibility of distal outcome findings in applied mixture modeling research.

## Pairwise comparisons

In applied research, it is standard practice to follow an omnibus test with post hoc pairwise comparisons when group-level differences are of substantive interest. For example, in a one-way analysis of variance with three groups, a significant omnibus *F* test is typically followed by three pairwise comparisons to determine which groups differ from one another. An analogous inferential structure arises in mixture modeling, where class-specific differences on distal outcomes are commonly evaluated using an omnibus Wald test, followed—if significant—by pairwise comparisons of class-specific means (Asparouhov, 2007).

Unlike single-test settings, pairwise distal outcome comparisons in mixture models constitute a family of related hypothesis tests, the size of which increases rapidly with the number of latent classes. These follow-up comparisons are not isolated statistical decisions. As in observed-group ANOVA settings, pairwise contrasts are typically statistically dependent because the same group- or class-specific means enter multiple comparisons. For example, in a three-class model, the Class 1 distal outcome mean contributes to both the Class 1 versus Class 2 and Class 1 versus Class 3 contrasts. As the number of classes and outcomes increases, these overlapping contrasts generate a large set of correlated tests within a single study. Multiplicity control is therefore needed because the comparison family as a whole increases the opportunity for false-positive findings and requires an explicit error-rate criterion, even when individual pairwise tests are statistically dependent rather than independent (Benjamini & Yekutieli, 2001; Blakesley et al., 2009; Glickman et al., 2014; Goeman & Solari, 2014; Mudge et al., 2012).

## Family-wise error rate (FWER)

One traditional approach to this problem is to control the family-wise error rate (FWER), defined a**s** the probability of making at least one Type I error within the family (Benjamini & Hochberg, 1995; Glickman et al., 2014). Under independence, the FWER increases rapidly as the number of comparisons grows, even when each individual test is conducted at a conventional significance level (e.g., α = .05). In mixture modeling applications, this escalation is particularly pronounced because the number of pairwise comparisons increases combinatorially with the number of latent classes, and distal outcome analyses are often conducted for multiple outcomes within the same study. As a result, unadjusted pairwise testing can substantially inflate false-positive findings and undermine the interpretability of reported class differences.

## Bonferroni correction

A commonly used approach for controlling the FWER is the Bonferroni correction (Bonferroni, 1936), which adjusts the per-comparison significance threshold by dividing the target family-wise α level by the number of tests conducted. This procedure guarantees strong control of the FWER and is straightforward to implement, making it a popular choice in applied research. However, because the Bonferroni correction does not account for correlations among tests and equally penalizes each comparison, it is widely regarded as overly conservative in settings involving many correlated tests. In the context of mixture modeling, this conservatism can lead to substantial losses in statistical power and an increased risk of Type II error, particularly when distal outcome differences are modest in magnitude (Perneger, 1998).

## Strengths and limitations

The primary strength of the Bonferroni correction lies in its simplicity and its strict control of false-positive findings. However, this same strictness represents its principal limitation: as the number of comparisons increases, the procedure rapidly becomes insensitive to meaningful effects. Consequently, while the Bonferroni correction may be appropriate in settings where false positives carry exceptionally high costs, alternative procedures that offer a more favorable balance between Type I and Type II error are often preferable for distal outcome analyses in mixture models.

## Holm–Bonferroni correction

The Holm–Bonferroni procedure (Holm, 1979) provides a sequentially rejective alternative to the standard Bonferroni correction for controlling the family-wise error rate. Rather than applying a single adjusted significance threshold to all tests, the Holm procedure orders observed *p*-values from smallest to largest and evaluates them against progressively less stringent critical values. This step-down approach retains strong control of the FWER while offering greater statistical power than the classical Bonferroni correction.

In applied settings, the Holm–Bonferroni procedure is guaranteed to reject at least as many hypotheses as the Bonferroni correction and is therefore uniformly more powerful (Eichstaedt et al., 2013; Levin, 1996). However, because it still targets strict FWER control, the Holm procedure can remain conservative when the number of comparisons is large or when tests are correlated—conditions that commonly arise in distal outcome analyses for mixture models. As a result, meaningful class differences may fail to reach significance even when consistent patterns are present across comparisons.

## Benjamini–Hochberg procedure

The Benjamini–Hochberg (BH) procedure (Benjamini & Hochberg, 1995) offers a conceptually distinct approach to multiple-comparison correction by controlling the false discovery rate (FDR), defined as the expected proportion of false positives among the set of rejected hypotheses. Rather than controlling the probability of making any false-positive inference within a family of tests, as in family-wise error-rate procedures, BH controls the expected proportion of false discoveries among statistically significant findings. This distinction is central to distal outcome analyses in mixture models because researchers often evaluate multiple pairwise comparisons across several latent classes or profiles, frequently across more than one distal outcome.

For applied distal outcome comparisons in mixture modeling, we recommend the BH procedure as the default multiplicity correction. This recommendation follows from the inferential structure of distal outcome reporting. In many applied mixture modeling studies, the analytic goal is to characterize the pattern, magnitude, and precision of differences across latent groups rather than to make a single isolated confirmatory decision. When several classes and outcomes are involved, strict family-wise error-rate procedures can become overly conservative, reducing power and obscuring substantively meaningful patterns of class differences. By controlling the FDR, BH provides a principled balance between false-positive control and sensitivity to systematic distal outcome differences.

## Advantages for distal outcome analyses in mixture models

The BH procedure is especially well suited to distal outcome analyses in mixture models for several reasons. First, it retains greater power than family-wise error-rate procedures as the number of pairwise comparisons increases, which is important when models include multiple latent classes and several distal outcomes. Second, the FDR framework aligns with the descriptive and comparative goals that often motivate person-centered research, where researchers seek to identify interpretable patterns of class differences rather than simply determine whether any single contrast is statistically significant. Third, BH produces a transparent error-rate interpretation: among the comparisons declared statistically significant, the expected proportion of false discoveries is controlled at the selected FDR threshold. This interpretation is useful for reporting because it allows researchers to describe which distal outcome differences remain robust after accounting for multiplicity without imposing an unnecessarily conservative standard that may suppress meaningful effects.

## Considerations and limitations

The BH procedure entails a different inferential interpretation than family-wise error-rate procedures, and its use should be accompanied by clear reporting of the selected FDR threshold and the family of tests to which the correction was applied. FDR control does not imply that no false positives are present among statistically significant comparisons; instead, it controls their expected proportion among rejected hypotheses. In the context of applied distal outcome reporting, this is a strength of the procedure rather than a limitation, because it provides explicit error-rate control while preserving sensitivity to the pattern of class differences.

Family-wise error-rate procedures may still be appropriate in narrowly confirmatory settings or when the consequence of even one false-positive comparison is unusually high. However, for the applied descriptive and comparative goals that commonly motivate distal outcome analyses in mixture modeling, BH provides the most appropriate default correction for multiple pairwise distal outcome comparisons. A worked example illustrating implementation of the BH procedure is provided in Supplement A.

## Effect size estimation for distal outcomes

Statistical significance alone provides limited information about the substantive importance of distal outcome differences across latent classes. Omnibus tests and pairwise comparisons indicate whether distal outcome means differ statistically, but they do not indicate how large those differences are or whether the observed pattern of differences is substantively meaningful. To complement hypothesis testing, effect size estimates are essential for quantifying the magnitude of distal outcome separation across latent groups and for facilitating interpretation across studies. In the context of mixture modeling, effect size estimation poses unique challenges because class membership is probabilistic, class sizes are often unequal, and class-specific distal outcome estimates are typically obtained through auxiliary-variable procedures rather than from directly observed groups.

Lanza et al. (2013) proposed an effect size measure, denoted ω (LTB-ω), that quantifies the overall association between a latent categorical variable and a continuous distal outcome. This measure represents a weighted dispersion of class-specific means around the grand mean, with weights proportional to class membership probabilities, and is expressed on the scale of Cohen’s *d* (Cohen, 1992). As such, LTB-ω provides a single interpretable summary of the extent to which a distal outcome varies across latent classes, independent of any specific pairwise comparison.

In the reporting workflow proposed here, LTB-ω serves as the global effect-size counterpart to the omnibus distal outcome test. The omnibus test evaluates whether the class-specific distal means are statistically distinguishable from one another; LTB-ω quantifies the overall magnitude of that separation. This distinction is important because statistically significant omnibus tests may correspond to small or substantively limited differences, whereas nontrivial global separation can be obscured when interpretation relies only on individual pairwise tests. Reporting LTB-ω therefore helps researchers characterize the overall strength of association between latent group membership and the distal outcome before turning to specific class-to-class comparisons.

In addition to global effect sizes, pairwise effect size estimates are necessary to interpret specific class-to-class differences following omnibus testing. For continuous distal outcomes, standardized mean differences, such as Cohen’s *d*, can be computed using class-specific means and variability estimates derived from standard mixture model output. Although mixture modeling software does not always report class-specific standard deviations directly, within-class variability is available in Mplus via the Model Estimated Covariances section of the residual output, that is, the covariance of the outcome with itself for each class, from which class-specific standard deviations can be obtained. Computational details are provided in Supplement B.

Global and pairwise effect sizes answer complementary questions. LTB-ω summarizes the overall magnitude of distal outcome separation across all latent classes, whereas pairwise Cohen’s *d* values quantify the magnitude of specific class-to-class differences. Reporting both prevents researchers from relying exclusively on statistical significance and supports a more complete interpretation of distal outcome results. Worked examples and computational details for effect size estimation are provided in Supplement B.

## Confidence intervals for distal outcome estimates

Confidence intervals provide essential information about the precision and stability of estimated distal outcome differences and should accompany both statistical tests and effect size estimates. In mixture modeling applications, confidence intervals are particularly valuable because class-specific estimates are subject to uncertainty arising from probabilistic class membership, unequal class sizes, and auxiliary-variable estimation. Reporting confidence intervals alongside point estimates allows readers to assess the range of plausible values for class differences and discourages over-interpretation of statistically significant results.

For distal outcome mean differences, confidence intervals can often be obtained directly from standard mixture modeling software. In Mplus, for example, confidence intervals for class-specific mean differences are available by requesting confidence interval output for model estimates, requiring no additional computation. Confidence intervals for standardized effect sizes, such as Cohen’s *d*, can be derived using established methods based on sample size and variability estimates (Hedges & Olkin, 2014).

Within the proposed reporting workflow, confidence intervals serve a different purpose than multiplicity-adjusted *p*-values. Adjusted *p*-values help determine which pairwise comparisons remain statistically distinguishable after accounting for multiple testing, whereas confidence intervals convey the precision and plausible magnitude of the estimated effects. This distinction is important in distal outcome reporting because a comparison may be statistically significant but imprecisely estimated, or nonsignificant but still compatible with substantively meaningful effects. Reporting confidence intervals therefore helps readers evaluate both inferential uncertainty and practical importance.

We recommend that researchers routinely report confidence intervals for both unstandardized mean differences and standardized effect size estimates in distal outcome analyses. When interpreted jointly with multiplicity-adjusted *p*-values, global effect sizes, and pairwise standardized effects, confidence intervals provide a more complete and transparent characterization of distal outcome differences across latent classes. Worked examples and computational details for confidence interval estimation are provided in Supplement C. Supplement C also includes a publication-style reporting template that illustrates how researchers can present distal outcome means, class-specific standard deviations, LTB-ω, pairwise mean differences, and standardized pairwise effects in a compact format suitable for applied manuscripts.

## Discussion

Distal outcome comparisons are among the most common and substantively consequential extensions of mixture models in applied behavioral research. Consistent with the targeted reporting-practice audit reported above, applied reports often rely on omnibus tests without systematic follow-up, conduct unadjusted pairwise comparisons across multiple latent classes, and omit global effect size estimates, pairwise standardized effects, and confidence intervals. These practices complicate interpretation and can inflate false-positive findings, particularly when distal outcome analyses involve many outcomes and many class-to-class comparisons. Consistent with broader reporting guidance emphasizing transparency and estimation-based inference (Appelbaum et al., 2018; Wasserstein & Lazar, 2016), the present paper provides a set of practical recommendations for strengthening distal outcome reporting in mixture modeling applications. The analytic sequence summarized in Table 1 provides a practical template for implementing these recommendations, highlighting the ordering of omnibus testing, follow-up comparisons, multiplicity control, global and pairwise effect size reporting, and confidence interval estimation in applied mixture modeling research.

This paper contributes a method-agnostic post-estimation framework focused on three core components of distal outcome inference: (a) explicitly defining families of pairwise comparisons and treating distal outcome follow-up testing as a multiple-comparisons problem; (b) applying multiplicity control procedures appropriate for settings involving many correlated tests, with BH recommended as the default procedure for applied distal outcome comparisons; and (c) routinely reporting global effect sizes, pairwise standardized effects, and confidence intervals alongside significance tests. Together, these practices improve interpretability by clarifying which class differences are robust to multiplicity adjustment, quantifying the magnitude of observed differences, and communicating uncertainty in estimated effects.

## Recommended reporting elements for distal outcome analyses

To promote consistent reporting and facilitate cumulative science, we recommend that distal outcome results in mixture modeling studies include the following elements for each outcome:

1. An omnibus test of distal outcome differences across classes (e.g., a Wald test)
2. Follow-up pairwise comparisons when omnibus tests indicate evidence of differences, with the family of comparisons clearly defined
3. A stated multiplicity control procedure, with BH recommended as the default for applied distal outcome comparisons, and the corresponding adjusted inference
4. A global effect size summarizing distal outcome separation across classes when appropriate (e.g., LTB-ω for continuous outcomes)
5. Pairwise standardized effect sizes (e.g., Cohen’s *d*) for **class-to-class** contrasts, and
6. Confidence intervals for both unstandardized differences and standardized effect sizes

For the applied descriptive and comparative goals that typically motivate distal outcome analyses in mixture modeling, we recommend BH as the default multiplicity correction because it provides a favorable balance between false-positive control and statistical power in settings involving many correlated comparisons. Family-wise error-rate procedures may still be appropriate for narrowly confirmatory settings or when the consequence of a single false-positive comparison is unusually high. Regardless of the correction method selected, transparent reporting of the chosen error-rate target, the selected threshold, and the definition of the comparison family is essential.

## Limitations and future directions

The present paper synthesizes recommendations from the broader multiple-testing and reporting literature and adapts them to distal outcome analyses in mixture models; it does not attempt to provide a comprehensive comparison of all available multiplicity procedures or to evaluate their operating characteristics under specific mixture modeling conditions. Future work could extend this contribution by using simulation studies tailored to common mixture modeling scenarios (e.g., varying class separation, class size imbalance, classification uncertainty, and correlation among outcomes) to evaluate the performance of alternative correction strategies for distal outcome inference. The software-agnostic Quarto/R workflow provided in Supplement D is intended as a practical post-estimation implementation aid, but future work could extend this contribution by developing dedicated software tools that automatically extract relevant quantities from mixture model output and streamline omnibus testing, multiplicity correction, effect size estimation, and confidence interval reporting across estimation platforms.

## Conclusion

Mixture models are frequently interpreted through patterns of distal outcome differences across classes, making distal outcome inference central to substantive conclusions drawn from latent class and profile solutions. Because these analyses naturally generate families of related hypothesis tests, rigorous and transparent reporting requires more than *p*-values from unadjusted pairwise comparisons. By integrating omnibus testing, BH-adjusted pairwise inference, global and pairwise effect size estimation, and confidence interval reporting, applied researchers can improve the interpretability, reproducibility, and practical usefulness of mixture modeling findings.

## Supplementary Information

Below is the link to the electronic supplementary material.

Supplementary file1 (PDF 1.68 MB)

Supplementary file2 (PDF 642 KB)

## Data Availability

Not applicable.

## Code availability

A reproducible Quarto/R post-estimation workflow for distal outcome reporting is provided in Supplement D. The corresponding Quarto source file and example materials are available at https://github.com/delwincarter/distal-outcomes-mixture-reporting.
