# Supplementary material for: Distal outcomes in mixture modeling: A guide for pairwise comparisons, multiplicity control, and effect size reporting
Source: Behav Res Methods. 2026 Aug 14;58(9):268. doi: 10.3758/s13428-026-03144-4 (PMC13476281; doi:10.3758/s13428-026-03144-4)
Supplement: Supplementary file 1 [file 13428_2026_3144_MOESM1_ESM.pdf]

Supplements A–C for "Distal Outcomes in Mixture Modeling: A Guide for Pairwise Comparisons, Multiplicity Control, and Effect Size Reporting"

AUTHOR
Delwin B. Carter

Supplement A

Multiplicity in Distal Outcome Pairwise Comparisons

A.1 Pairwise Comparisons and Families of Tests

Distal outcome analyses in mixture models typically involve evaluating differences in outcome means across multiple latent classes or profiles. When more than two classes are present, these differences are examined through a set of pairwise comparisons that collectively form a *family* of hypothesis tests. This structure is analogous to post hoc testing following a significant omnibus test in classical analysis of variance (ANOVA). In the mixture modeling context, class differences are commonly evaluated using an omnibus Wald test (Asparouhov, 2007), followed—if significant—by pairwise comparisons of class-specific outcome means.

When multiple pairwise tests are conducted, the probability of making at least one false-positive inference increases. This risk motivates the use of formal multiplicity control procedures in distal outcome analyses (Blakesley et al., 2009; Goeman & Solari, 2014; Mudge et al., 2012).

A.2 Omnibus Tests for Distal Outcome Differences

Prior to conducting pairwise comparisons of class-specific distal outcome means, researchers should evaluate whether there is evidence of any overall differences across latent classes. In mixture modeling applications, this is typically accomplished using an omnibus Wald test that assesses the null hypothesis that all class-specific distal outcome means are equal (Asparouhov, 2007). This test serves as the appropriate gatekeeper for follow-up pairwise comparisons and defines the family of hypothesis tests to which multiplicity correction procedures apply.

In practice, omnibus tests for distal outcomes are not always automatically requested or clearly labeled in mixture modeling software output. As a result, applied researchers may inadvertently proceed directly to pairwise comparisons without first establishing an overall class effect. Explicitly requesting and reporting an omnibus test is therefore a critical step in distal outcome inference.

The following example illustrates how an omnibus Wald test for distal outcome differences can be implemented in practice.

Implementation in Mplus

When distal outcomes are included using stepwise approaches (e.g., ML three-step or BCH), omnibus tests of class-specific mean differences can be implemented using the MODEL TEST command in Mplus. Conceptually, the omnibus Wald test evaluates a set of linear constraints equating distal outcome means across all classes.

For example, for a distal outcome *d* with three latent classes, an omnibus test can be specified as:

Mplus syntax for omnibus Wald testing

MODEL CONSTRAINT:

NEW(d1 d2 d3);

MODEL TEST:

0 = d1 - d2;

0 = d1 - d3;

Here, *d*1, *d*2, and *d*3 represent the class-specific distal outcome means defined in the model, and the MODEL TEST statement jointly evaluates whether these means are equal across classes.

This specification jointly tests whether the distal outcome means for all classes are equal. The resulting Wald chi-square test statistic and associated degrees of freedom appear in the MODEL TEST RESULTS section of the Mplus output.

If the omnibus test is statistically significant, researchers may proceed to examine specific class-to-class differences using pairwise comparisons. If the omnibus test is not significant, follow-up pairwise testing is generally not warranted.

A.3 Family-Wise Error Rate (FWER)

The *family-wise error rate* (FWER) is defined as the probability of making at least one Type I error within a family of hypothesis tests (Benjamini & Hochberg, 1995; Glickman et al., 2014). Under independence, the FWER is given by Equation (A1):

$$\mathrm{FWER} = 1 - (1 - \alpha)^n$$

where α is the per-test significance level and n is the number of tests conducted.

Worked Examples

- **Single test**
  With α = .05 and n = 1:

$$\mathrm{FWER} = 1 - (1 - .05)^1 = .05$$

- **Three pairwise comparisons**
  With α = .05 and n = 3:

$$\mathrm{FWER} = 1 - (1 - .05)^3 = .143$$

- **Six latent classes (15 comparisons)**
  With α = .05 and n = 15:

$$\mathrm{FWER} = 1 - (1 - .05)^{15} = .536$$

These examples illustrate that the probability of at least one false-positive finding increases rapidly as the number of pairwise comparisons grows, a common scenario in mixture modeling applications involving multiple latent classes and outcomes.

A.4 Bonferroni Correction

The Bonferroni correction controls the FWER by dividing the desired family-wise significance level by the number of tests conducted (Bonferroni, 1936), as shown in Equation (A2).

$$\alpha_{\text{adjusted}} = \frac{\alpha_{\text{original}}}{n} \tag{A2}$$

Example

Consider a model with three latent classes and one distal outcome, resulting in three pairwise comparisons. Applying Equation (A2) with three comparisons yields an adjusted significance threshold of α = .0167.

$$\alpha_{\text{adjusted}} = \frac{.05}{3} = .0167$$

If the observed p-values are:

- Class 1 vs. 2: p = .04
- Class 2 vs. 3: p = .005
- Class 1 vs. 3: p = .03

Only the second comparison would be considered statistically significant under the Bonferroni correction.

A.5 Holm–Bonferroni Procedure

The Holm–Bonferroni procedure (Holm, 1979) is a sequentially rejective method that controls the FWER while offering greater power than the classical Bonferroni correction. Observed p-

values are ranked from smallest to largest and compared against progressively less stringent critical values defined in Equation (A3).

$$\alpha_i = \frac{\alpha}{n - i + 1} \tag{A3}$$

where i denotes the rank of the p-value. Table A1 illustrates the application of Equation (A3) to a set of three ordered p-values.

Table A1

Holm–Bonferroni Sequential Testing Example

Rank	Comparison	p-value	Critical α
1	Class 2 vs. 3	.005	.0167
2	Class 1 vs. 3	.03	.025
3	Class 1 vs. 2	.04	.05

Note. Critical α values are computed using the Holm–Bonferroni procedure with familywise α = .05.

The smallest p-value is significant at its adjusted level. The second-ranked p-value exceeds its threshold, and all subsequent tests are therefore considered non-significant.

The Holm–Bonferroni procedure is guaranteed to reject at least as many hypotheses as the Bonferroni correction and is uniformly more powerful (Eichstaedt et al., 2013; Levin, 1996), though it remains conservative when the number of comparisons is large.

A.6 Benjamini–Hochberg Procedure

The Benjamini–Hochberg (BH) procedure (Benjamini & Hochberg, 1995) controls the *false discovery rate* (FDR), defined as the expected proportion of false positives among rejected hypotheses. Unlike FWER-controlling procedures, the BH method explicitly balances false-positive control with statistical power.

The procedure operates by:

1. Ranking p-values in ascending order
2. Comparing each p-value to a rank-specific critical value $(i/n)\cdot q$, where q is the target FDR level
3. Declaring all p-values up to the largest significant rank as significant

Table A2

Benjamini–Hochberg False Discovery Rate Example (q = .05)

	Comparisons									
	1 vs. 4	3 vs. 4	1 vs. 5	2 vs. 4	1 vs. 2	4 vs. 5	2 vs. 3	2 vs. 5	1 vs. 3	3 vs. 5
p-values	.003	.004	.005	.018	.022	.032	.037	.048	.077	.087
*d * i/n*	.005	.010	.015	.020	.025	.030	.035	.040	.045	.050
Significant?	Yes	Yes	Yes	Yes	Yes	No	No	No	No	No

Note. Critical values are computed as (i/n)·q, where *i* is the rank of the p-value, *n* is the total number of comparisons, and *q* is the target false discovery rate.

In this example, five comparisons are retained as statistically significant under FDR control at $q = .05$.

A.7 Summary

This supplement illustrates how distal outcome analyses in mixture models naturally generate families of related hypothesis tests and demonstrates how different multiplicity control procedures operate in practice. Bonferroni and Holm–Bonferroni procedures provide strict control of the family-wise error rate, whereas the Benjamini–Hochberg procedure controls the false discovery rate and typically offers greater power in settings involving many correlated comparisons.

Supplement B

Effect Size Computation for Distal Outcomes in Mixture Models

B.1 Global Effect Size for Distal Outcomes: LTB-ω

Lanza et al. (2013) proposed an effect size measure, denoted ω (LTB-ω), to quantify the overall association between a latent categorical variable and a distal outcome. This measure is expressed on the scale of Cohen's *d* (Cohen, 1992) and provides a single summary of the extent to which a distal outcome varies across latent classes or profiles. LTB-ω is applicable to both categorical and continuous distal outcomes; because continuous outcomes are most common in applied mixture modeling research, only the continuous-outcome formulation is presented here (see Lanza et al., 2013, for categorical outcomes).

The formula for calculating the effect size LTB-ω for continuous outcome variables is given in Equation (B1).

$$LTB - \omega = \sqrt{\sum_{c=1}^{K} \pi_c (\mu_c - \bar{\mu})^2} \qquad \text{(B1)}$$

where

$\boldsymbol{\pi}_c$ is the estimated probability of membership in class *c*,

μ_c is the class-specific mean of the distal outcome, and

$\overline{\mu}$ is the grand mean of the distal outcome across classes.

Conceptually, LTB-ω represents a probability-weighted dispersion of class-specific means around the grand mean, with weights proportional to class membership probabilities.

B.2 Worked Example: Global Effect Size (LTB-ω)

To illustrate the computation of LTB-ω, consider an example adapted from Moore et al. (2024). A mixture model of covitality identified four ordered latent profiles: High Covitality (HC), Moderate-High Covitality (MHC), Moderate-Low Covitality (MLC), and Low Covitality (LC). Estimated class membership probabilities and distal outcome means for life satisfaction were as follows:

- $\boldsymbol{\pi}_{HC\sim = .21,}$ μ_{HC}~ = 5.40
- $\boldsymbol{\pi}_{MHC}$ = .41, μ_{MHC} = 4.78
- $\boldsymbol{\pi}_{MLC}$ = .30, μ_{MLC} = 3.79
- $\boldsymbol{\pi}_{LC}$ = .08, μ_{LC} = 2.89

The grand mean of life satisfaction across classes was $\bar{\mu} = 4.19$.

Table B1 presents the intermediate quantities used to compute LTB-ω.

Table B1

Calculation of LTB-ω for a Continuous Distal Outcome with Four Latent Classes

Class	Outcome Variable M	Class Membership Probability	μ - $\bar{\mu}$	$(\mu - \bar{\mu})^2$	Pr * $(\mu - \bar{\mu})^2$	LTB-ω
HC	5.40	0.21	0.91	0.84	0.18	
MHC	4.78	0.41	0.29	0.09	0.04	
MLC	3.79	0.30	-0.70	0.48	0.15	
LC	2.89	0.08	-1.60	2.56	0.19	
Grand Mean	**4.90**					
Sum					**0.55**	
$\sqrt{\text{Sum}}$						**0.74**

Note. HC = High Covitality; MHC = Moderate–High Covitality; MLC = Moderate–Low Covitality; LC = Low Covitality. μ denotes the class-specific distal outcome mean, and the grand mean is computed across all classes using class membership probabilities.

The resulting LTB-ω value of 0.74 indicates a large overall association between latent profile membership and life satisfaction, according to conventional benchmarks for Cohen's *d* (Cohen, 1992).

B.3 Pairwise Effect Sizes: Cohen's *d*

In addition to global effect sizes, pairwise standardized mean differences are often useful for interpreting specific class-to-class differences following omnibus testing. For continuous distal outcomes, Cohen's *d* for a comparison between classes 1 and 2 is computed using Equation (B2).

$$d = \frac{\mu_1 - \mu_2}{SD_{\text{pooled}}} \tag{B2}$$

The pooled standard deviation required for Equation (B2) is defined in Equation (B3).

$$SD_{\text{pooled}} = \sqrt{\frac{SD_1^2 + SD_2^2}{2}} \tag{B3}$$

To compute the quantities in Equations (B2)–(B3), researchers require class-specific means (μ_k) and class-specific within-class standard deviations (SD_k) for the distal outcome. Many mixture modeling programs report the class-specific mean structure directly, and they also report the class-specific within-class variance of the distal outcome *(i.e., the covariance of the outcome with itself, COV(Y, Y | C = k))* in the residual output under *Model Estimated Covariances* for each class. When the within-class variance is available, the corresponding class-specific standard deviation is obtained using Equation (B4).

$$SD_k = \sqrt{\text{Var}(Y \mid C = k)} \tag{B4}$$

These class-specific standard deviations can then be inserted into Equation (B3) to compute pooled standard deviations for any desired pairwise comparison, and subsequently into Equation (B2) to compute Cohen's *d*.

B.4 Worked Example: Obtaining Class-Specific Standard Deviations from Residual Output

This worked example illustrates how the class-specific standard deviations required for Equations (B2)–(B3) can be obtained directly from standard model output. The distal outcome in this example is life satisfaction (BMLSS). For each latent class, the within-class variance of BMLSS is taken from the *Model Estimated Covariances* section of the residual output, where the covariance of BMLSS with itself corresponds to Var(BMLSS | $C = k$). Class-specific standard deviations are then computed using Equation (B4) and are presented in Table B2.

Table B2

Class-Specific Means, Variances, and Standard Deviations for BMLSS

Class	Mean (μ)	Var(BMLSS | $C = k$)	SD(BMLSS | $C = k$)
1	5.417	0.246	0.496
2	4.796	0.366	0.605
3	3.803	0.741	0.861
4	2.902	1.074	1.036

Note. Variances are obtained from the *Model Estimated Covariances* section of the residual output for each latent class. Standard deviations are computed as the square root of the class-specific variance (Equation B4).

B.5 Pairwise Cohen's *d* Calculations

Using the class-specific standard deviations obtained from the residual output (Section B.4), pooled standard deviations were computed for each pairwise class comparison using Equation (B3). These pooled standard deviations were then used in Equation (B2) to compute Cohen's *d* values for each pairwise mean difference. Results are presented in Table B3.

Table B3

Pairwise Standardized Mean Differences (Cohen's d)

Comparison	Mean Diff	Pooled SD	Cohen's d
HC − MHC	0.621	0.554	1.12
MHC − MLC	0.993	0.747	1.33
MLC − LC	0.901	0.955	0.94
HC − MLC	1.614	0.701	2.30
MHC − LC	1.894	0.848	2.23
HC − LC	2.515	0.811	3.10

Note. Cohen's *d* represents standardized mean differences computed using pooled standard deviations derived from recovered class-specific standard deviations.

These results illustrate how pairwise effect sizes can complement omnibus and global effect size measures by quantifying the magnitude of specific class differences on a standardized scale.

Supplement C

Confidence Interval Computation for Distal Outcome Effect Sizes

C.1 Confidence Intervals for Distal Outcome Mean Differences

Confidence intervals provide critical information about the precision of estimated distal outcome differences and complement both significance testing and effect size estimation. In mixture modeling applications, confidence intervals are particularly informative because class-specific estimates reflect uncertainty due to probabilistic class membership and unequal class sizes.

For unstandardized distal outcome mean differences, confidence intervals can be obtained directly from standard mixture modeling software. In Mplus, confidence intervals for class-specific mean differences are available by requesting confidence interval output (e.g., via the CINTERVAL option), which returns 90%, 95%, and 99% confidence intervals for estimated parameters. No additional computation is required.

C.2 Confidence Intervals for Cohen's *d*

Confidence intervals for standardized mean differences (Cohen's *d*) can be computed using established methods based on large-sample normal approximations (Hedges & Olkin, 2014). A 95% confidence interval for Cohen's *d* is given by:

$$d \pm 1.96 \times SE(d) \tag{C1}$$

where $SE(d)$ denotes the standard error of the Cohen's d.

For independent groups, the standard error of Cohen's d can be approximated as:

$$SE(d) = \sqrt{\frac{N_1 + N_2}{N_1 N_2} + \frac{d^2}{2(N_1 + N_2)}} \tag{C2}$$

where N_1 and N_2 are the sample sizes of the two classes being compared, and d is the estimated standardized mean difference.

This formulation allows confidence intervals for Cohen's d to be computed using quantities readily available from mixture model output and class assignment summaries.

C.3 Worked Example: Confidence Intervals for Distal Outcome Effect Sizes

Using the pairwise comparisons presented in Supplement B, confidence intervals were computed for both unstandardized mean differences and standardized effect sizes. Results are shown in Table C1.

Table C1

Pairwise Mean Differences and Cohen's d with 95% Confidence Intervals

Comparison	Mean Difference	95% CI (Mean Difference)	Cohen's d	95% CI (d)
HC − MHC	0.62	[0.57, 0.67]	1.16	[1.10, 1.22]
MHC − MLC	0.99	[0.94, 1.05]	1.65	[1.59, 1.71]
MLC − LC	0.90	[0.78, 1.03]	0.84	[0.71, 0.90]
HC − MLC	1.61	[1.55, 1.67]	2.59	[2.50, 2.67]
MHC − LC	1.90	[1.78, 2.01]	1.76	[1.67, 1.86]
HC − LC	2.51	[2.40, 2.63]	2.31	[2.20, 2.43]

Note. HC = High Covitality; MHC = Moderate–High Covitality; MLC = Moderate–Low Covitality; LC = Low Covitality. Confidence intervals correspond to 95% intervals for both unstandardized mean differences and standardized effect sizes.

These results illustrate how confidence intervals complement point estimates by conveying the range of plausible values for both unstandardized and standardized distal outcome differences. Narrow confidence intervals indicate greater precision in estimated class differences, whereas wider intervals signal greater uncertainty.

C.4 Interpretation Considerations

Confidence intervals for Cohen's d should be interpreted as reflecting uncertainty in the estimated standardized mean difference, not the distribution of individual outcomes. When reported alongside multiplicity-adjusted p-values and effect size estimates, confidence intervals

provide a transparent and interpretable summary of distal outcome differences across latent classes.

C.5 Publication-Style Reporting Example

The worked examples in Supplements B and C illustrate how global and pairwise effect size estimates can be computed from quantities available in standard mixture-model output. In applied manuscripts, these quantities must ultimately be reported in a format that is compact enough for publication while still communicating the information needed for interpretation. Table C2 provides an example of a publication-style table for reporting distal outcome comparisons across latent profiles.

The table summarizes, for each distal outcome, the global LTB-ω value, profile-specific means and standard deviations, pairwise mean differences, and Cohen's *d* values for each class-to-class comparison. This format allows readers to evaluate both the overall magnitude of distal outcome separation across profiles and the magnitude of specific pairwise differences without requiring separate tables for means, global effects, and standardized contrasts.

Table C2

Publication-style table for reporting distal outcome means, global effect sizes, pairwise mean differences, and standardized pairwise effects

Outcome	LTB-ω	Profile	μ	*SD*		Comparisons					
						μ_1-μ_2	μ_2-μ_3	μ_3-μ_4	μ_1-μ_3	μ_2-μ_4	μ_1-μ_4
Self- Report Grades	.48	1. High	5.91	1.40	μ_{diff}	0.34	0.58	0.64	0.91	1.21	1.55
		2. Moderate-High	5.58	0.76							
		3. Moderate-Low	5.00	0.81	Cohen's *d*	0.23	0.36	0.35	0.57	0.78	1.01
		4. Low	4.37	0.50							
School Connectedness	.43	1. High	4.48	1.48	μ_{diff}	0.37	0.57	0.38	0.94	0.95	1.32
		2. Moderate-High	4.11	0.58							
		3. Moderate-Low	3.54	0.72	Cohen's *d*	0.58	0.98	0.50	1.21	1.55	1.72
		4. Low	3.17	0.60							
Distress	.36	1. High	1.36	1.75	μ_{diff}	-0.24	-0.57	-0.14	-0.82	-0.71	-0.96
		2. Moderate-High	1.60	0.75							
		3. Moderate-Low	2.18	0.79	Cohen's *d*	0.32	0.76	0.17	1.02	0.95	1.15
		4. Low	2.32	0.86							
Life Satisfaction	.79	1. High	5.83	1.96	μ_{diff}	0.61	0.99	0.91	1.61	1.90	2.52
		2. Moderate-High	5.22	0.79							
		3. Moderate-Low	4.23	0.92	Cohen's *d*	1.08	1.44	1.08	2.34	2.77	3.91
		4. Low	3.32	1.04							

Note. LTB-ω = Lanza, Tan, and Bray omega. μ = profile-specific distal outcome mean. *SD* = profile-specific standard deviation. μ_{diff} = pairwise mean difference. Cohen's *d* = standardized pairwise mean difference. Response categories for the scales were as follows: self-report grades = 1 = mostly F's to 8 = mostly A's; school connectedness = 1 = strongly disagree to 5 = strongly agree; distress = 1 = not at all true to 4 = very much true; life satisfaction = 1 = very dissatisfied to 6 = very satisfied

References

Asparouhov, T. (2007, November 16). *Wald test of mean equality for potential latent class predictors in mixture modeling*. Muthén & Muthén, Mplus. https://www.statmodel.com/download/MeanTest1.pdf

Benjamini, Y., & Hochberg, Y. (1995). Controlling the false discovery rate: A practical and powerful approach to multiple testing. *Journal of the Royal Statistical Society: Series B (Methodological)*, *57*(1), 289-300. https://doi.org/10.1111/j.2517-6161.1995.tb02031.x

Blakesley, R. E., Mazumdar, S., Dew, M. A., Houck, P. R., Tang, G., Reynolds, C. F., & Butters, M. A. (2009). Comparisons of methods for multiple hypothesis testing in neuropsychological research. *Neuropsychology*, *23*(2), 255-264. https://doi.org/10.1037/a0012850

Bonferroni, C. E. (1936). *Teoria statistica delle classi e calcolo delle probabilità*. Pubblicazioni del R Istituto Superiore di Scienze Economiche e Commerciali di Firenze.

Cohen, J. (1992). Statistical power analysis. *Current Directions in Psychological Science*, *1*(3), 98-101. https://doi.org/10.1111/1467-8721.ep10768783

Eichstaedt, K. E., Kovatch, K., & Maroof, D. A. (2013). A less conservative method to adjust for familywise error rate in neuropsychological research: The holm's sequential Bonferroni procedure. *NeuroRehabilitation*, *32*(3), 693-696. https://doi.org/10.3233/nre-130893

Glickman, M. E., Rao, S. R., & Schultz, M. R. (2014). False discovery rate control is a recommended alternative to bonferroni-type adjustments in health studies. *Journal of Clinical Epidemiology*, *67*(8), 850-857. https://doi.org/10.1016/j.jclinepi.2014.03.012

Goeman, J. J., & Solari, A. (2014). Multiple hypothesis testing in genomics. *Statistics in Medicine*, *33*(11), 1946-1978. https://doi.org/10.1002/sim.6082

Hedges, L. V., & Olkin, I. (2014). *Statistical methods for meta-analysis*. Academic Press.

Holm, S. (1979). A simple sequentially rejective multiple test procedure. *Scandinavian Journal of Statistics*, *6*(2), 65–70. https://www.jstor.org/stable/4615733

Lanza, S. T., Tan, X., & Bray, B. C. (2013). Latent Class Analysis With Distal Outcomes: A Flexible Model-Based Approach. *Structural Equation Modeling: A Multidisciplinary Journal, 20*(1), 1-26. https://doi.org/10.1080/10705511.2013.742377

Levin, B. (1996). On the holm, Simes, and Hochberg multiple test procedures. *American Journal of Public Health*, *86*(5), 628-629. https://doi.org/10.2105/ajph.86.5.628

Moore, S. A., Carter, D., Kim, E. K., Furlong, M. J., Nylund-Gibson, K., & Dowdy, E. (2024). Adolescents' Covitality patterns: Relations with student demographic characteristics and proximal academic and mental health outcomes. *School Mental Health*, *16*(2), 550-565. https://doi.org/10.1007/s12310-024-09663-9

Mudge, J. F., Baker, L. F., Edge, C. B., & Houlahan, J. E. (2012). Setting an optimal α that minimizes errors in null hypothesis significance tests. *PLoS ONE*, *7*(2), e32734. https://doi.org/10.1371/journal.pone.0032734
