# Supplementary material for: Distal outcomes in mixture modeling: A guide for pairwise comparisons, multiplicity control, and effect size reporting
Source: Behav Res Methods. 2026 Aug 14;58(9):268. doi: 10.3758/s13428-026-03144-4 (PMC13476281; doi:10.3758/s13428-026-03144-4)
Supplement: Supplementary file 2 [file 13428_2026_3144_MOESM2_ESM.pdf]

Supplement D for “Distal Outcomes in Mixture Modeling: A Guide for Pairwise Comparisons, Multiplicity Control, and Effect Size Reporting”: Reproducible R Workflow for Distal Outcome Reporting

AUTHOR
Delwin B. Carter

Overview

Supplement D provides a reproducible post-estimation workflow for distal outcome reporting in mixture models. The workflow is designed for analyses in which a latent class or latent profile solution has already been selected and distal outcome comparisons have already been estimated in mixture-modeling software.

The workflow connects the reporting components described in Supplements A–C in a single reproducible workflow. Given class-specific probabilities, distal outcome means, within-class variances, class-specific sample sizes, and pairwise comparison *p*-values, the workflow computes class-specific standard deviations, the weighted grand mean, LTB-ω, pairwise mean differences, pooled standard deviations, Cohen’s *d*, standard errors and confidence intervals for Cohen’s *d*, and multiplicity-adjusted *p*-values.

Scope

This supplement is a post-estimation reporting workflow. It assumes that the mixture model has already been estimated, the class solution has already been selected, and the distal outcome comparisons have already been obtained from model output.

The workflow does not estimate the mixture model, determine the number of classes, conduct the omnibus Wald test, or extract values automatically from Mplus or other software. Instead, it provides a reproducible reporting layer that converts model output into the derived quantities needed for distal outcome reporting.

The workflow is intentionally software-agnostic. Researchers can use values obtained from Mplus, Latent GOLD, SAS-based LCA workflows, StepMix/StepMixR, R-based mixture-modeling packages, or other software, provided that the necessary post-estimation quantities are available. The purpose of the supplement is to help researchers move from model output to transparent reporting of the quantities emphasized in the tutorial.

The workflow is not limited to the four-class example shown below. The pairwise comparison structure is generated from the class labels supplied in the input chunk, so the same code can be

applied to solutions with any number of latent classes, profiles, trajectories, or transitions, provided that the input vectors have matching lengths and the number of pairwise p-values matches the number of class-to-class comparisons.

The tables in this supplement are designed to make the computational process transparent. They show how values from model output are converted into the quantities recommended in the manuscript. They are not intended to prescribe a single required table format for publication. A publication-style reporting example is provided separately in Supplement C.

Repository and interactive workflow

Repository archive. The complete Quarto/R source for this reproducible post-estimation workflow is publicly available in the **GitHub repository**.

Interactive HTML workflow. In addition to this journal-formatted PDF supplement, a rendered HTML version of the workflow is available online. The HTML version contains the same post-estimation workflow, example inputs, computations, and outputs, with inline code, tables, and section-level navigation for ease of methodological review and reuse.

Access the interactive workflow.

Required inputs

The workflow requires six inputs: `class_label`, `class_prob`, `class_mean`, `class_variance`, `class_n`, and `pairwise_p`.

`class_label` contains the class or profile labels. These labels are user-defined and should match the class names used in the distal outcome interpretation.

`class_prob` contains the estimated class membership probabilities. These values come from the selected mixture model output and should be entered as proportions, not percentages. For example, enter `.21`, not `21`.

`class_mean` contains the class-specific distal outcome means. These values come from the distal outcome model output.

`class_variance` contains the class-specific within-class variances for the distal outcome. These values are needed to recover the class-specific standard deviations used in the pairwise Cohen's d calculations. In Mplus, they are not reported as class-specific standard deviations. They are found in the residual output under Model Estimated Covariances for each class.

For a given distal outcome, the required value is the covariance of that outcome with itself within each class-specific covariance matrix. For example, for BMLSS, enter the BMLSS-with-BMLSS value from each class-specific covariance matrix. Users should request residual output when

estimating the model; otherwise, this section may not appear in the output. Enter variances directly; the workflow computes standard deviations internally.

`class_n` contains the class-specific sample sizes or estimated class counts. These values come from the class enumeration or classification summary output.

`pairwise_p` contains the pairwise comparison *p*-values from the distal outcome model output. These values are used to compute Bonferroni-adjusted, Holm-adjusted, and Benjamini–Hochberg/FDR-adjusted *p*-values.

Input vector structure

For a four-class model, the input vectors follow this structure:

`class_label <- c("Class 1", "Class 2", "Class 3", "Class 4")`
`class_prob <- c(prob_1, prob_2, prob_3, prob_4)`
`class_mean <- c(mean_1, mean_2, mean_3, mean_4)`
`class_variance <- c(var_1, var_2, var_3, var_4)`
`class_n <- c(n_1, n_2, n_3, n_4)`
`pairwise_p <- c(p_12, p_13, p_14, p_23, p_24, p_34)`

For models with a different number of classes, add or remove entries from each vector. The workflow determines the number of classes from `class_label` and checks that the number of pairwise *p*-values matches the required number of class-to-class comparisons.

For four classes, the pairwise *p*-value order is:

`p_12` = Class 1 vs. Class 2
`p_13` = Class 1 vs. Class 3
`p_14` = Class 1 vs. Class 4
`p_23` = Class 2 vs. Class 3
`p_24` = Class 2 vs. Class 4
`p_34` = Class 3 vs. Class 4

Load Packages

This workflow uses `gt` to format the output tables.

```
library(gt)
```

BMLSS application

The workflow is applied to the BMLSS distal outcome example used in Supplements B and C. The example uses four classes: HC, MHC, MLC, and LC.

The input values are entered below and carried through the workflow to generate diagnostic tables and post-estimation output. Because the substantive BMLSS pairwise *p*-values are uniformly small, the example *p*-values used here are instructional values selected to show how Bonferroni, Holm–Bonferroni, and Benjamini–Hochberg adjustments can lead to different inferential decisions when pairwise tests fall near common decision thresholds.

BMLSS input values

The values below come from the BMLSS distal outcome example used in Supplements B and C. To apply the workflow to another distal outcome model, replace the values in this input chunk with values from your own model output. All values in the example are taken from the BMLSS worked example except the pairwise *p*-values. The pairwise *p*-values are instructional values selected to fall near common decision thresholds so that the workflow can illustrate how Bonferroni, Holm–Bonferroni, and Benjamini–Hochberg adjustments can lead to different inferential decisions.

```
# Replace these example values with values from your own distal outcome analysis.

class_label <- c("HC", "MHC", "MLC", "LC")

class_prob <- c(.21, .41, .30, .08)

class_mean <- c(5.417, 4.796, 3.803, 2.902)

class_variance <- c(0.246, 0.366, 0.741, 1.074)

class_n <- c(210, 410, 300, 80)

pairwise_p <- c(.003, .009, .018, .026, .041, .073)
```

Input checks

Before computing the derived quantities, the workflow checks whether the input vectors are internally consistent.

The checks verify that the class-level vectors contain the same number of entries, that the number of pairwise *p*-values matches the number of class-to-class comparisons, that class

probabilities are entered as proportions and sum approximately to 1, that variances and sample sizes are positive, and that pairwise *p*-values fall between 0 and 1.

These checks are included to identify data-entry errors before the post-estimation output is generated.

Helper functions

The following chunk defines the helper functions used by the workflow. Do not edit this chunk. Run it once before generating the output tables.

```
check_distal_inputs <- function(class_label,
                                class_prob,
                                class_mean,
                                class_variance,
                                class_n,
                                pairwise_p,
                                prob_tolerance = .01) {

  K <- length(class_label)

  class_lengths <- c(
    class_label = length(class_label),
    class_prob = length(class_prob),
    class_mean = length(class_mean),
    class_variance = length(class_variance),
    class_n = length(class_n)
  )

  if (length(unique(class_lengths)) != 1) {
    stop(
      "All class-level input vectors must have the same length.\n",
      "Observed lengths:\n",
      paste(names(class_lengths), class_lengths, sep = " = ", collapse = "\n")
    )
  }

  expected_pairwise <- choose(K, 2)

  if (length(pairwise_p) != expected_pairwise) {
    stop(
      "The number of pairwise p-values must equal choose(K, 2).\n",
      "K = ", K, "\n",
      "Expected pairwise p-values = ", expected_pairwise, "\n",
```

```r
      "Observed pairwise p-values = ", length(pairwise_p)
    )
  }

  if (any(class_prob < 0, na.rm = TRUE)) {
    stop("Class probabilities must be nonnegative.")
  }

  if (abs(sum(class_prob) - 1) > prob_tolerance) {
    stop(
      "Class probabilities should sum approximately to 1.\n",
      "Observed sum = ", round(sum(class_prob), 4), "\n",
      "If your output gives percentages, enter them as proportions. For example, enter
         .21 instead of 21."
    )
  }

  if (any(class_variance <= 0, na.rm = TRUE)) {
    stop("Class-specific variances must be positive.")
  }

  if (any(class_n <= 0, na.rm = TRUE)) {
    stop("Class-specific sample sizes must be positive.")
  }

  if (any(pairwise_p < 0 | pairwise_p > 1, na.rm = TRUE)) {
    stop("Pairwise p-values must be between 0 and 1.")
  }

  invisible(TRUE)
}


make_class_table <- function(class_label,
                             class_prob,
                             class_mean,
                             class_variance,
                             class_n) {

  class_sd <- sqrt(class_variance)

  data.frame(
    class = class_label,
    class_probability = class_prob,
    class_mean = class_mean,
    class_variance = class_variance,
```

```r
    class_sd = class_sd,
    class_n = class_n,
    stringsAsFactors = FALSE
  )
}


compute_ltb_omega <- function(class_prob,
                              class_mean) {

  weighted_grand_mean <- sum(class_prob * class_mean)

  ltb_omega <- sqrt(
    sum(class_prob * (class_mean - weighted_grand_mean)^2)
  )

  list(
    weighted_grand_mean = weighted_grand_mean,
    ltb_omega = ltb_omega
  )
}


make_pairwise_table <- function(class_label,
                                class_mean,
                                class_variance,
                                class_n,
                                pairwise_p,
                                alpha = .05) {

  K <- length(class_label)

  pair_index <- combn(K, 2)

  class_sd <- sqrt(class_variance)

  class_1_index <- pair_index[1, ]
  class_2_index <- pair_index[2, ]

  mean_difference <- class_mean[class_1_index] - class_mean[class_2_index]

  pooled_sd <- sqrt(
    (class_sd[class_1_index]^2 + class_sd[class_2_index]^2) / 2
  )

  cohens_d <- mean_difference / pooled_sd
```

```r
  se_d <- sqrt(
    ((class_n[class_1_index] + class_n[class_2_index]) /
       (class_n[class_1_index] * class_n[class_2_index])) +
      ((cohens_d^2) / (2 * (class_n[class_1_index] + class_n[class_2_index])))
  )

  d_ci_lower <- cohens_d - 1.96 * se_d
  d_ci_upper <- cohens_d + 1.96 * se_d

  p_bonferroni <- p.adjust(pairwise_p, method = "bonferroni")
  p_holm <- p.adjust(pairwise_p, method = "holm")
  p_bh <- p.adjust(pairwise_p, method = "BH")

  alpha <- .05

  data.frame(
    comparison = paste(class_label[class_1_index], "vs.", class_label[class_2_index]),
    class_1 = class_label[class_1_index],
    class_2 = class_label[class_2_index],
    mean_1 = class_mean[class_1_index],
    mean_2 = class_mean[class_2_index],
    mean_difference = mean_difference,
    pooled_sd = pooled_sd,
    cohens_d = cohens_d,
    se_d = se_d,
    d_ci_lower = d_ci_lower,
    d_ci_upper = d_ci_upper,
    p_unadjusted = pairwise_p,
    p_bonferroni = p_bonferroni,
    p_holm = p_holm,
    p_bh_fdr = p_bh,
    sig_unadjusted = ifelse(pairwise_p < alpha, "Yes", "No"),
    sig_bonferroni = ifelse(p_bonferroni < alpha, "Yes", "No"),
    sig_holm = ifelse(p_holm < alpha, "Yes", "No"),
    sig_bh_fdr = ifelse(p_bh < alpha, "Yes", "No"),
    stringsAsFactors = FALSE
  )
}


round_numeric_columns <- function(data, digits = 3) {

  numeric_columns <- vapply(data, is.numeric, logical(1))

  data[numeric_columns] <- lapply(
```

```
    data[numeric_columns],
    round,
    digits = digits
  )

  data
}
```

Input validation

This step applies the input checks to the values entered above. If an input has the wrong length or an impossible value, the workflow stops and returns an error message identifying the issue.

```
check_distal_inputs(
  class_label = class_label,
  class_prob = class_prob,
  class_mean = class_mean,
  class_variance = class_variance,
  class_n = class_n,
  pairwise_p = pairwise_p
)
```

Class-specific quantities table

This table displays the class-specific quantities used in the workflow. Standard deviations are computed from the class-specific within-class variances entered in the input chunk.

```
class_table <- make_class_table(
  class_label = class_label,
  class_prob = class_prob,
  class_mean = class_mean,
  class_variance = class_variance,
  class_n = class_n
)

class_table |>
  gt() |>
  tab_header(
    title = "Class-Specific Distal Outcome Quantities",
    subtitle = "Example values for BMLSS"
  ) |>
```

```r
  cols_label(
    class = "Class",
    class_probability = "Class probability",
    class_mean = "Mean",
    class_variance = "Variance",
    class_sd = gt::md("*SD*"),
    class_n = gt::md("*N*")
  ) |>
  fmt_number(
    columns = c(class_probability, class_mean, class_variance, class_sd),
    decimals = 3
  ) |>
  fmt_number(
    columns = class_n,
    decimals = 0
  ) |>
  cols_align(
    align = "center",
    columns = class
  ) |>
  cols_align(
    align = "right",
    columns = c(class_probability, class_mean, class_variance, class_sd, class_n)
  ) |>
  tab_source_note(
    source_note = gt::md("Note. *SD* is computed from the class-specific within-class
         variance.")
  )
```

Class-Specific Distal Outcome Quantities

Example values for BMLSS

Class	Class probability	Mean	Variance	*SD*	*N*
HC	0.210	5.417	0.246	0.496	210
MHC	0.410	4.796	0.366	0.605	410
MLC	0.300	3.803	0.741	0.861	300
LC	0.080	2.902	1.074	1.036	80

Note. *SD* is computed from the class-specific within-class variance.

Comparison structure table

The workflow uses the class labels to determine how many pairwise comparisons are needed. For the BMLSS example, there are four classes and six pairwise comparisons.

```
K <- length(class_label)

number_pairwise_comparisons <- choose(K, 2)

comparison_summary <- data.frame(
  Quantity = c("Number of classes", "Number of pairwise comparisons"),
  Value = c(K, number_pairwise_comparisons)
)

comparison_summary |>
  gt() |>
  tab_header(
    title = "Model Comparison Structure"
  ) |>
  cols_align(
    align = "left",
    columns = Quantity
  ) |>
  cols_align(
    align = "center",
    columns = Value
  )
```

Model Comparison Structure

Quantity	Value
Number of classes	4
Number of pairwise comparisons	6

Pairwise *p*-value order table

This table displays the comparison order generated by `combn()`. The *p*-values entered in `pairwise_p` are assigned to comparisons in this exact order.

Pairwise p-Value Order		
Order used to match entered p-values to class comparisons		
Position	Comparison	p-value
1	HC vs. MHC	0.003
2	HC vs. MLC	0.009
3	HC vs. LC	0.018
4	MHC vs. MLC	0.026
5	MHC vs. LC	0.041
6	MLC vs. LC	0.073

Global distal outcome summary table

This table reports the weighted grand mean and the global LTB-ω effect size for the distal outcome.

```
ltb_results <- compute_ltb_omega(
  class_prob = class_prob,
  class_mean = class_mean
)

weighted_grand_mean <- ltb_results$weighted_grand_mean

ltb_omega <- ltb_results$ltb_omega

ltb_summary <- data.frame(
  Quantity = c("Weighted grand mean", "LTB-ω"),
  Value = c(weighted_grand_mean, ltb_omega)
)

ltb_summary |>
  gt() |>
  tab_header(
    title = "Global Distal Outcome Summary",
    subtitle = "Weighted grand mean and LTB-ω"
  ) |>
  fmt_number(
```

```
    columns = Value,
    decimals = 3
  ) |>
  cols_align(
    align = "left",
    columns = Quantity
  ) |>
  cols_align(
    align = "right",
    columns = Value
  )
```

Global Distal Outcome Summary	
Weighted grand mean and LTB-ω	
Quantity	Value
Weighted grand mean	4.477
LTB-ω	0.750

LTB-ω calculation components table

The global LTB-ω value summarizes how far the class-specific means are spread around the weighted grand mean. This table displays each class's contribution to that calculation. It is included as a diagnostic table to verify that the global effect size is computed from the intended class probabilities and class-specific means.

```
ltb_table <- data.frame(
  class = class_label,
  class_probability = class_prob,
  class_mean = class_mean,
  deviation_from_grand_mean = class_mean - weighted_grand_mean,
  squared_deviation = (class_mean - weighted_grand_mean)^2,
  weighted_squared_deviation = class_prob * (class_mean - weighted_grand_mean)^2,
  stringsAsFactors = FALSE
)

ltb_table |>
  gt() |>
  tab_header(
    title = "LTB-ω Calculation Components",
```

```
    subtitle = "Probability-weighted dispersion of class-specific means"
  ) |>
  cols_label(
    class = "Class",
    class_probability = "Class probability",
    class_mean = "Mean",
    deviation_from_grand_mean = "Mean − weighted grand mean",
    squared_deviation = "Squared deviation",
    weighted_squared_deviation = "Weighted squared deviation"
  ) |>
  fmt_number(
    columns = c(
      class_probability,
      class_mean,
      deviation_from_grand_mean,
      squared_deviation,
      weighted_squared_deviation
    ),
    decimals = 3
  ) |>
  cols_align(
    align = "center",
    columns = class
  ) |>
  cols_align(
    align = "right",
    columns = c(
      class_probability,
      class_mean,
      deviation_from_grand_mean,
      squared_deviation,
      weighted_squared_deviation
    )
  ) |>
  tab_source_note(
    source_note = paste0(
      "Note. Weighted grand mean = ",
      round(weighted_grand_mean, 3),
      "; LTB-ω = ",
      round(ltb_omega, 3),
      "."
    )
  )
```

LTB-ω Calculation Components					
Probability-weighted dispersion of class-specific means					
Class	Class probability	Mean	Mean − weighted grand mean	Squared deviation	Weighted squared deviation
HC	0.210	5.417	0.940	0.884	0.186
MHC	0.410	4.796	0.319	0.102	0.042
MLC	0.300	3.803	−0.674	0.454	0.136
LC	0.080	2.902	−1.575	2.481	0.198

Note. Weighted grand mean = 4.477; LTB-ω = 0.75.

Pairwise diagnostic table

This table displays the full set of pairwise class comparisons generated by the workflow. It includes pairwise mean differences, pooled standard deviations, Cohen's *d*, standard errors for Cohen's *d*, 95% confidence intervals for Cohen's *d*, and multiplicity-adjusted *p*-values.

This table is intended as a diagnostic output. It shows the full set of quantities computed before selecting the smaller set of columns used in the post-estimation results table.

```r
pairwise_table <- make_pairwise_table(
  class_label = class_label,
  class_mean = class_mean,
  class_variance = class_variance,
  class_n = class_n,
  pairwise_p = pairwise_p,
  alpha = .05
)

pairwise_display <- pairwise_table[
  ,
  c(
    "comparison",
    "mean_difference",
    "pooled_sd",
    "cohens_d",
    "se_d",
    "d_ci_lower",
```

```r
    "d_ci_upper",
    "p_unadjusted",
    "p_bonferroni",
    "p_holm",
    "p_bh_fdr"
  )
]

pairwise_display |>
  gt() |>
  tab_header(
    title = "Pairwise Distal Outcome Comparisons",
    subtitle = gt::md("Full diagnostic table for effect-size and *p*-value adjustment
         quantities")
  ) |>
  cols_label(
    comparison = "Comparison",
    mean_difference = "Mean difference",
    pooled_sd = "Pooled SD",
    cohens_d = gt::md("Cohen's *d*"),
    se_d = gt::md("SE(*d*)"),
    d_ci_lower = "95% CI lower",
    d_ci_upper = "95% CI upper",
    p_unadjusted = gt::md("Unadjusted *p*"),
    p_bonferroni = gt::md("Bonferroni *p*"),
    p_holm = gt::md("Holm *p*"),
    p_bh_fdr = gt::md("BH/FDR *p*")
  ) |>
  fmt_number(
    columns = c(
      mean_difference,
      pooled_sd,
      cohens_d,
      se_d,
      d_ci_lower,
      d_ci_upper,
      p_unadjusted,
      p_bonferroni,
      p_holm,
      p_bh_fdr
    ),
    decimals = 3
  ) |>
  cols_align(
    align = "left",
    columns = comparison
```

```
  ) |>
tab_options(
  table.font.size = px(11)
) |>
  cols_align(
    align = "right",
    columns = c(
      mean_difference,
      pooled_sd,
      cohens_d,
      se_d,
      d_ci_lower,
      d_ci_upper,
      p_unadjusted,
      p_bonferroni,
      p_holm,
      p_bh_fdr
    )
  ) |>
  tab_source_note(
    source_note = "Note. This table is intended as a diagnostic output showing all
        pairwise quantities computed by the workflow."
  )
```

Pairwise Distal Outcome Comparisons

Full diagnostic table for effect-size and *p*-value adjustment quantities

Comparison	Mean difference	Pooled SD	Cohen's *d*	SE(*d*)	95% CI lower	95% CI upper	Unadjusted *p*	Bonferroni *p*	Holm *p*	BH/FDR *p*
HC vs. MHC	0.621	0.553	1.123	0.091	0.945	1.300	0.003	0.018	0.018	0.018
HC vs. MLC	1.614	0.702	2.298	0.115	2.072	2.523	0.009	0.054	0.045	0.027
HC vs. LC	2.515	0.812	3.096	0.184	2.735	3.456	0.018	0.108	0.072	0.036
MHC vs. MLC	0.993	0.744	1.335	0.084	1.170	1.499	0.026	0.156	0.078	0.039
MHC vs. LC	1.894	0.849	2.232	0.142	1.955	2.509	0.041	0.246	0.082	0.049
MLC vs. LC	0.901	0.953	0.946	0.130	0.690	1.201	0.073	0.438	0.082	0.073

Note. This table is intended as a diagnostic output showing all pairwise quantities computed by the workflow.

Post-Estimation Results Table

The table below brings together the main results computed from the post-estimation quantities entered above. It includes the pairwise mean difference, Cohen's *d*, the confidence interval for Cohen's *d*, the BH-adjusted *p*-value, and the corresponding significance decision for each class-to-class comparison. This table is intended to help researchers check and document the quantities produced by the workflow. Researchers may use or adapt these results when preparing their own manuscript tables. A publication-style example of how distal outcome results can be presented in an applied manuscript is provided in Supplement C.

```
primary_table <- pairwise_table[
  ,
  c(
    "comparison",
    "mean_difference",
    "cohens_d",
    "d_ci_lower",
    "d_ci_upper",
    "p_bh_fdr",
    "sig_bh_fdr"
  )
]

primary_table |>
  gt() |>
  tab_header(
    title = "Post-Estimation Pairwise Distal Outcome Results",
    subtitle = gt::md("Mean differences, standardized effects, confidence intervals,
         and BH/FDR-adjusted *p*-values")
  ) |>
  cols_label(
    comparison = "Comparison",
    mean_difference = "Mean difference",
    cohens_d = gt::md("Cohen's *d*"),
    d_ci_lower = "95% CI lower",
    d_ci_upper = "95% CI upper",
    p_bh_fdr = gt::md("BH/FDR *p*"),
    sig_bh_fdr = "Significant after BH/FDR"
  ) |>
  fmt_number(
    columns = c(
      mean_difference,
      cohens_d,
      d_ci_lower,
      d_ci_upper,
      p_bh_fdr
    ),
```

```
    decimals = 3
  ) |>
  tab_options(
  table.font.size = px(12)
) |>
  cols_align(
    align = "left",
    columns = comparison
  ) |>
  cols_align(
    align = "right",
    columns = c(
      mean_difference,
      cohens_d,
      d_ci_lower,
      d_ci_upper,
      p_bh_fdr
    )
  ) |>
  cols_align(
    align = "center",
    columns = sig_bh_fdr
  ) |>
  tab_source_note(
  source_note = "Note. BH/FDR = Benjamini-Hochberg false discovery rate adjustment.
         Significant after BH/FDR indicates whether the comparison is statistically
         significant after BH/FDR adjustment at alpha = .05."
)
```

Post-Estimation Pairwise Distal Outcome Results

Mean differences, standardized effects, confidence intervals, and BH/FDR-adjusted *p*-values

Comparison	Mean difference	Cohen's *d*	95% CI lower	95% CI upper	BH/FDR *p*	Significant after BH/FDR
HC vs. MHC	0.621	1.123	0.945	1.300	0.018	Yes
HC vs. MLC	1.614	2.298	2.072	2.523	0.027	Yes
HC vs. LC	2.515	3.096	2.735	3.456	0.036	Yes
MHC vs. MLC	0.993	1.335	1.170	1.499	0.039	Yes
MHC vs. LC	1.894	2.232	1.955	2.509	0.049	Yes
MLC vs. LC	0.901	0.946	0.690	1.201	0.073	No

Note. BH/FDR = Benjamini–Hochberg false discovery rate adjustment. Significant after BH/FDR indicates whether the comparison is statistically significant after BH/FDR adjustment at alpha = .05.

Output notes

The unadjusted *p*-values are the original pairwise *p*-values entered from the mixture-model output. The adjusted *p*-values show how the same pairwise comparisons change under Bonferroni, Holm, and Benjamini–Hochberg/FDR correction.

Cohen's *d* is signed. A positive value indicates that the first class listed in the comparison has a higher distal outcome mean than the second class. A negative value indicates that the first class listed has a lower distal outcome mean than the second class.

Session information

```r
sessionInfo()
```

```
R version 4.6.1 (2026-06-24 ucrt)
Platform: x86_64-w64-mingw32/x64
Running under: Windows 11 x64 (build 26200)

Matrix products: default
  LAPACK version 3.12.1

locale:
[1] LC_COLLATE=English_United States.utf8
[2] LC_CTYPE=English_United States.utf8
[3] LC_MONETARY=English_United States.utf8
[4] LC_NUMERIC=C
[5] LC_TIME=English_United States.utf8

time zone: America/Los_Angeles
tzcode source: internal

attached base packages:
[1] stats     graphics  grDevices utils     datasets  methods   base

other attached packages:
[1] gt_1.3.0

loaded via a namespace (and not attached):
 [1] vctrs_0.7.3       cli_3.6.6         knitr_1.51        rlang_1.3.0
 [5] xfun_0.60         otel_0.2.0        generics_0.1.4    jsonlite_2.0.0
 [9] litedown_0.10     glue_1.8.1        markdown_2.0      htmltools_0.5.9
[13] sass_0.4.10       rmarkdown_2.31    evaluate_1.0.5    tibble_3.3.1
[17] base64enc_0.1-6   fastmap_1.2.0     yaml_2.3.12       lifecycle_1.0.5
```

```
[21] compiler_4.6.1    dplyr_1.2.1       fs_2.1.0          htmlwidgets_1.6.4
[25] pkgconfig_2.0.3   rstudioapi_0.19.0 digest_0.6.39     R6_2.6.1
[29] tidyselect_1.2.1  pillar_1.11.1     commonmark_2.0.0  magrittr_2.0.5
[33] tools_4.6.1       withr_3.0.3       xml2_1.6.0
```
